# Spatiotemporal distribution and environmental influences of severe fever with thrombocytopenia syndrome in Shandong Province, China

**DOI:** 10.1186/s12879-023-08899-1

**Published:** 2023-12-20

**Authors:** Qing Duan, Xueying Tian, Bo Pang, Yuwei Zhang, Chuanhao Xiao, Mingxiao Yao, Shujun Ding, Xiaomei Zhang, Xiaolin Jiang, Zengqiang Kou

**Affiliations:** 1 Infectious Disease Prevention and Control Section, Shandong Center for Disease Control and Prevention, Jinan, 250014 China; 2 Chinese Field Epidemiology Training Program, Chinese Center for Disease Control and Prevention, Beijing, 100050 China

**Keywords:** Severe fever with thrombocytopenia syndrome, Spatiotemporal distribution, Environmental influences, Spatiotemporal scan statistic, Maximum entropy model

## Abstract

**Background:**

Severe fever with thrombocytopenia syndrome (SFTS) is an emerging infectious disease discovered in China in 2009. The purpose of this study was to describe the spatiotemporal distribution of SFTS and to identify its environmental influencing factors and potential high-risk areas in Shandong Province, China.

**Methods:**

Data on the SFTS incidence from 2010 to 2021 were collected. Spatiotemporal scan statistics were used to identify the time and area of SFTS clustering. The maximum entropy (MaxEnt) model was used to analyse environmental influences and predict high-risk areas.

**Results:**

From 2010 to 2021, a total of 5705 cases of SFTS were reported in Shandong. The number of SFTS cases increased yearly, with a peak incidence from April to October each year. Spatiotemporal scan statistics showed the existence of one most likely cluster and two secondary likely clusters in Shandong. The most likely cluster was in the eastern region, from May to October 2021. The first secondary cluster was in the central region, from May to October 2021. The second secondary cluster was in the southeastern region, from May to September 2020. The MaxEnt model showed that the mean annual wind speed, NDVI, cattle density and annual cumulative precipitation were the key factors influencing the occurrence of SFTS. The predicted risk map showed that the area of high prevalence was 28,120 km^2^, accounting for 18.05% of the total area of the province.

**Conclusions:**

The spatiotemporal distribution of SFTS was heterogeneous and influenced by multidimensional environmental factors. This should be considered as a basis for delineating SFTS risk areas and developing SFTS prevention and control measures.

**Supplementary Information:**

The online version contains supplementary material available at 10.1186/s12879-023-08899-1.

## Introduction

An emerging infectious disease known as severe fever with thrombocytopenia syndrome (SFTS) was first identified in rural central China in 2009 [1]. Its aetiological pathogen is SFTS virus (SFTSV), which belongs to the genus *Phlebovirus* in the order *Bunyavirales* of the family *Phenuiviridae,* and is officially named *Dabie bandavirus* [2]. The clinical manifestations of SFTS are diverse and include fever, nausea, vomiting, anorexia, headache, fatigue, myalgia, diarrhoea, abdominal pain, and enlarged lymph nodes [3, 4]. In mainland China, the number of SFTS cases and the geographical scope involved have increased yearly. From 2011 to 2021, a total of 18,902 confirmed SFTS cases were reported, and the case fatality rate was 5.11% [5]. The researchers claimed that the reported mortality rate for SFTS may be low, as a topical study showed that the mortality rate for SFTS was as high as 16.2% in areas with a high prevalence [6]. SFTS has become a widely prevalent infectious disease in the Asia-Pacific region, and cases of SFTS have been reported in Japan, Korea, Thailand, Vietnam, and the United Arab Emirates, in addition to China [7–11]. Some SFTS-like cases have also been found in the United States and Australia [8, 12]. Due to the growing threat, SFTS was listed as one of nine emerging diseases prioritized for research and development by the World Health Organization (WHO) in 2017 and as one of the top 10 priority infectious diseases in 2018 [13, 14]. However, despite the seriousness and widespread prevalence of SFTS, no specific drug or vaccine for SFTS has been developed in humans to date [15, 16].

SFTS is a typical tick-borne infectious disease. SFTSV has been detected in *Haemaphysalis longicornis, Rhipicephalus microplus, Hyalomma asiaticum, Haemaphysalis concinna, Amblyomma testudinarium* and *Ixodes nipponensis* [17]. Previous research found a high prevalence of SFTSV infection in ticks of 11.1% [18]. Tick bites are the primary route of SFTSV infection in humans, but a small number of infections are caused by person-to-person transmission. From 1996 to 2019, a cumulative total of 27 human-to-human SFTS events were reported in China and South Korea, with exposure to patient body fluids, vomitus, and aerosols being the main risk factors [19]. In addition, antibodies or nucleic acids to SFTSV have been found in several animals, such as pigs, sheep, cattle, chickens, dogs, and cats [20–23]. As in humans, these animals are at high risk of infection once bitten by a tick carrying SFTSV.

Environmental factors can affect SFTSV survival, reproduction, and tick growth dynamics and directly affect human outdoor activities [24]. Transmission kinetic modelling showed that cutting off the transmission route from the environment to humans had the greatest impact on SFTS prevention and control, demonstrating that the environment plays an important role in the occurrence of SFTS [25]. To date, studies have surveyed the effects of environmental factors on SFTSV infection by constructing the MaxEnt model, generalized additive model, and distributed lag nonlinear model. The results showed that the occurrence of SFTS was related to barometric pressure, temperature, relative humidity, precipitation and sunshine duration [26–28]. Among the commonly available models, the MaxEnt model is more widely used, and it is often used to explore regional and environmental risk factors for the spread of naturally focused diseases. For example, the MaxEnt model combined with geographic information systems (GIS) has been applied to predict the distribution of multiple types of infectious diseases, such as African swine fever, Japanese encephalitis, schistosomiasis and scrub typhus [29–32].

In this study, the spatiotemporal clusters of SFTS cases in Shandong from 2010 to 2021 were analysed using spatiotemporal scan statistics. The MaxEnt model was used to predict the potential risk areas and to identify the environmental factors that have an impact on SFTS occurrence. The present findings are expected to guide public health workers in developing more targeted prevention strategies, which are important for promoting SFTS prevention and control.

## Materials and methods

### Study area

**Fig. 1:**
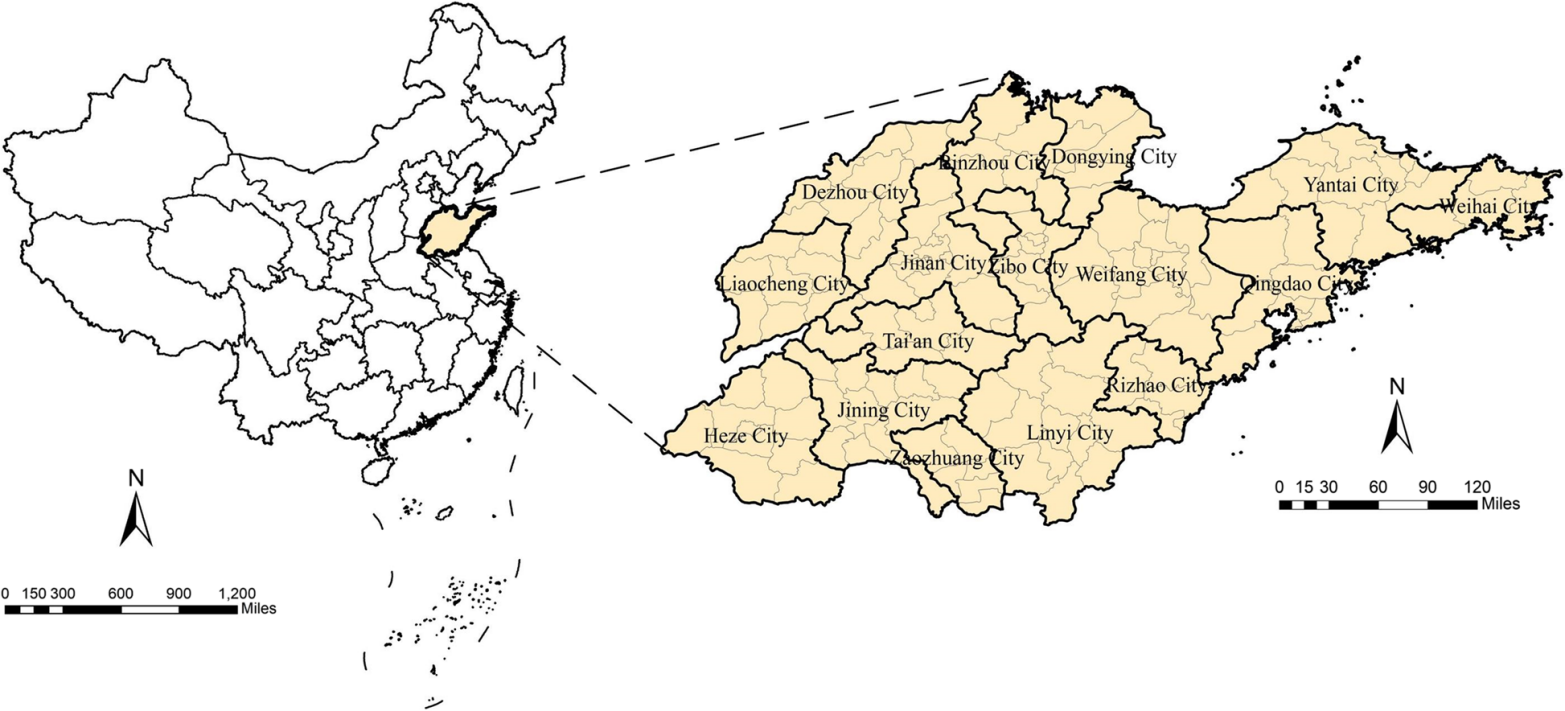
Map of Shandong Province in China

Shandong (34°22.9′N - 38°24.01′N, 114°47.5′E - 122°42.3′E) is an eastern coastal province of China and includes 16 cities and 136 counties. It has an east–west length of 721.03 km and a north–south length of 437.28 km, with a land area of 155,800 km2. With a resident population of over 100 million, Shandong ranks second in China [13] (Fig. 1).

### Data collection

Data on SFTS cases were collected from the infectious disease surveillance module of the China Information System for Disease Control and Prevention (CISDCP). The gathered information included age, sex, occupation, case type, date of diagnosis, reporting area and living address. The population data for each year were obtained from the basic information module of the CISDCP.

Climate data were obtained from the China Meteorological Data Sharing Service System (http://data.cma.cn/). Topographical data and socioeconomic data were collected from the Resource and Environment Science and Data Center (http://www.resdc.cn/). Livestock data were acquired from Livestock Geo-Wiki (http://livestock.geo-wiki.org/home-2/) (Table 1). All variables were transformed into a uniform ASCII raster format with a 1 km × 1 km spatial resolution.

**Table 1:** Explanation of environmental variables and their contribution to the MaxEnt model

Category	Abbreviation	Variable	Excluded	Percentage contribution (%)
Climate	EVP	Yearly average evaporation (mm)	Yes	–
	GST	Yearly average ground surface temperature (°C)	Yes	–
	PRS	Yearly average atmospheric pressure (hPa)	Yes	–
	RHU	Yearly average relative humidity (%)	No	4.5
	SSD	Yearly average sunshine duration (h)	Yes	–
	TEM	Yearly average air temperature (°C)	No	7.9
	WIN	Yearly average wind speed (m/s)	No	35.3
	PRE	Yearly cumulative precipitation (mm)	No	11.6
Topographical	ALT	Altitude (m)	No	6.3
	NDVI	Normalized difference vegetation index	No	14.8
Socioeconomic	GDP	Gross domestic product (10^4^ RMB/person)	No	0.2
	NLI	Nighttime light index	No	4.5
Livestock	Chicken_d	Chicken density (chickens/km^2^)	No	0.7
	Duck_d	Duck density (ducks/km^2^)	Yes	–
	Cattle_d	Cattle density (cattles/km^2^)	No	12.2
	Goat_d	Goat density (goats/km^2^)	No	1.2
	Sheep_d	Sheep density (sheep/km^2^)	No	0.8
	Pig_d	Pig density (pigs/km^2^)	Yes	–

### The spatiotemporal scan statistic

Spatiotemporal clustering of SFTS from January 2010 to December 2021 was identified using SatScan 9.5 software. SatScan is the most widely used spatial scan statistics software. According to different research scales, scan statistics can be categorized into three types: temporal, spatial and spatiotemporal scan statistics. Spatiotemporal scan statistical analysis was chosen for this study because it identifies both spatial location and specific features at a particular point in time. When setting the parameters, a discrete Poisson distribution model was selected for scanning, and the scan window was set to cylindrical. The maximum space size was defined as 20%, and the maximum time size was defined as 6 months. The logarithmic likelihood ratio (LLR) was constructed by theoretical incidence and actual incidence, and a statistical test was carried out. Statistically significant differences were considered when the *p* value was less than 0.05. The analysis was visualized using ArcGIS 10.7 software.

### The maximum entropy model

**Fig. 2:**
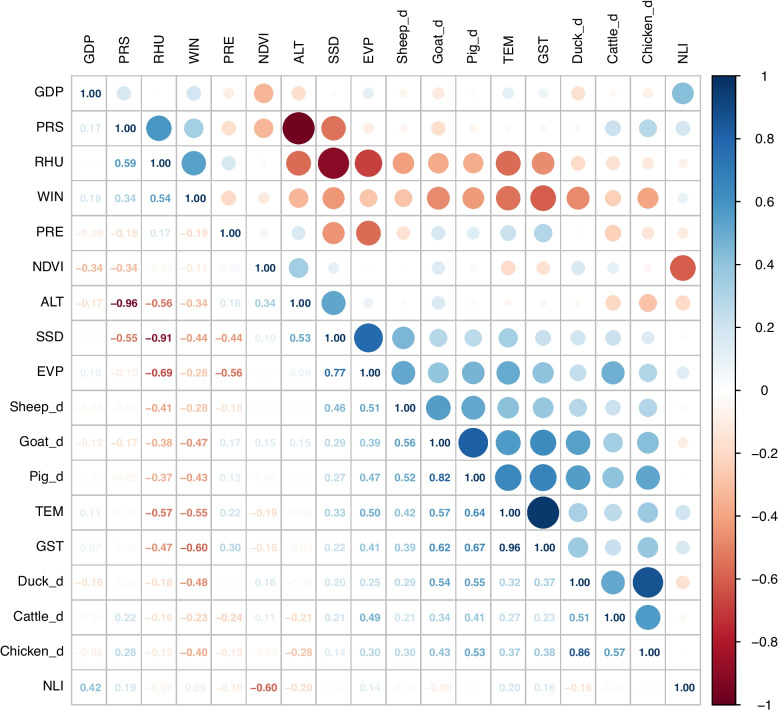
Correlation of environmental variables. GDP: Gross domestic product; PRS: Yearly average atmospheric pressure; RHU: Yearly average relative humidity; WIN: Yearly average wind speed; PRE: Yearly cumulative precipitation; NDVI: Normalized difference vegetation index; ALT: Altitude; SSD: Yearly average sunshine duration; EVP: Yearly average evaporation; Sheep_d: Sheep density; Goat_d: Goat density; Pig_d: Pig density; TEM: Yearly average air temperature; GST: Yearly average ground surface temperature; Duck_d: Duck density; Cattle_d: Cattle density; Chicken_d: Chicken density; NLI: Nighttime light index

The MaxEnt model is a machine learning method that is applied to exist-only datasets to model the potential distribution of a species and was originally used to estimate the probability distribution of a species. It is now widely used for the spatial prediction of infectious diseases. Using the MaxEnt model, it is possible to identify environmental influences that affect the distribution of disease and predict hotspots of incidence. Before constructing the model, the correlation coefficient between variables was calculated using cross-correlation analysis (Fig. 2). To avoid multicollinearity, only one variable was selected among those with a correlation coefficient > 0.7. Ultimately, 6 unsuitable variables were excluded, and the remaining 12 variables were included in the model (Table 1). In addition, the collection of patient locations may be influenced by diagnostic differences, resulting in overly dense data on the distribution of disease in specific regions, which may lead to overfitting of the study results. To avoid this problem, we filtered the distribution location data using ENMTools 3.4 software to remove data with possible errors or too high of a distribution density to avoid analysis errors and overfitting. Finally, 3440 valid positions were obtained.

When setting parameters, approximately 25% of the samples were randomly selected as the test dataset and 75% as the training dataset. The final result was an average of 10 runs on the same sample. The maximum number of iterations was set to 500, and the maximum number of background points was set to 10,000. The logical output format of the prediction map for the selected probability values was 0 (unsuitable) to 1 (suitable) to visualize the potential risk. The default prevalence parameter was set to 0.5 as the risk threshold to distinguish between potentially present regions and potentially absent regions. Response curves were plotted to elucidate the relationship between each variable and occurrence. The relative importance of different variables was assessed by calculating the percentage contribution of each variable using jackknife analysis. Variables with contribution percentages > 10% were considered to be significantly associated with the occurrence of SFTS. The accuracy of the model was evaluated using the area under the receiver operating characteristic curve (AUC). In general, AUC values < 0.7 indicated low model accuracy, 0.7–0.9 indicated accurate models, and > 0.9 indicated high accuracy. The risk prediction map was divided into nonendemic, low-prevalence, medium-prevalence and high-prevalence zones by the natural breakpoint method. The analysis was performed using MaxEnt 3.4.1 software and visualized using ArcGIS 10.7 software.

## Results

### Descriptive results

**Fig. 3:**
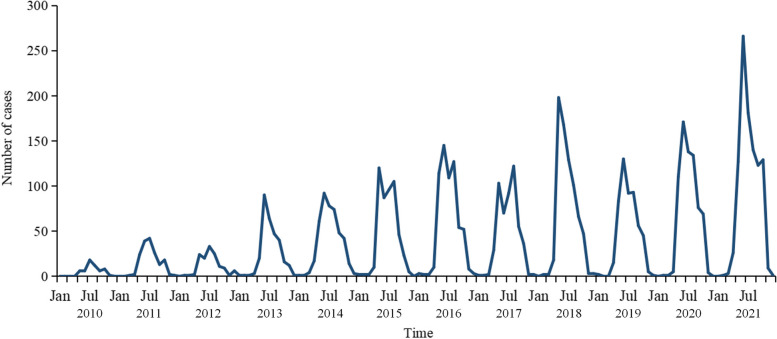
Number of SFTS cases per month in Shandong Province, China, 2010–2021

From 2010 to 2021, a total of 5705 SFTS cases were reported in Shandong Province, including 561 deaths, with a case fatality rate of 9.83%. The number of cases increased from 57 cases in 2010 to 1006 cases in 2021, showing a rapid upward trend. The occurrence of SFTS had a distinct seasonal pattern, with the number of cases starting to increase rapidly in April each year, reaching a peak from June to August, and dropping to a low after October (Fig. 3).

### The spatiotemporal scan statistic

**Fig. 4:**
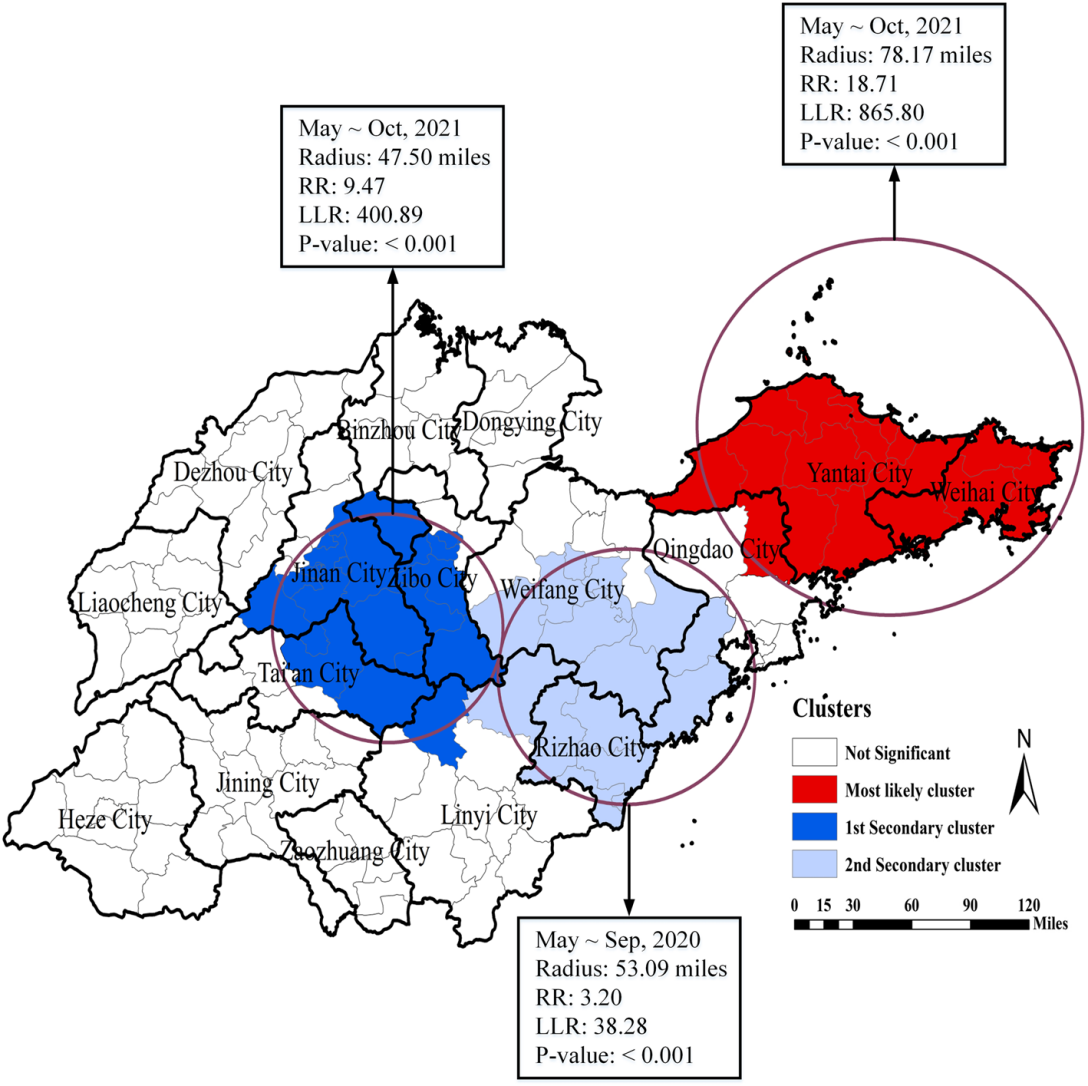
Spatiotemporal clusters of SFTS cases in Shandong Province, China, 2010–2021

Spatiotemporal scan analysis showed a nonrandom distribution of SFTS cases in Shandong Province from 2010 to 2021. One most likely cluster and two secondary likely clusters were detected. The most likely cluster covered northeastern Qingdao, the whole area of Yantai and the whole area of Weihai from May to October 2021 (RR = 18.71, LLR = 865.80, *p* < 0.01). The first secondary cluster included southeastern Jinan, southern Zibo, eastern Tai’an, northern Linyi and southern Binzhou from May to October 2021 (RR = 9.47, LLR = 400.89, *p* < 0.01). The second secondary cluster covered southern Qingdao, southern Weifang, northern Linyi and the whole area of Rizhao from May to September 2020 (RR = 3.20, LLR = 38.28, *p* < 0.01) (Fig. 4, Supplementary Table 1).

### The MaxEnt model

**Fig. 5:**
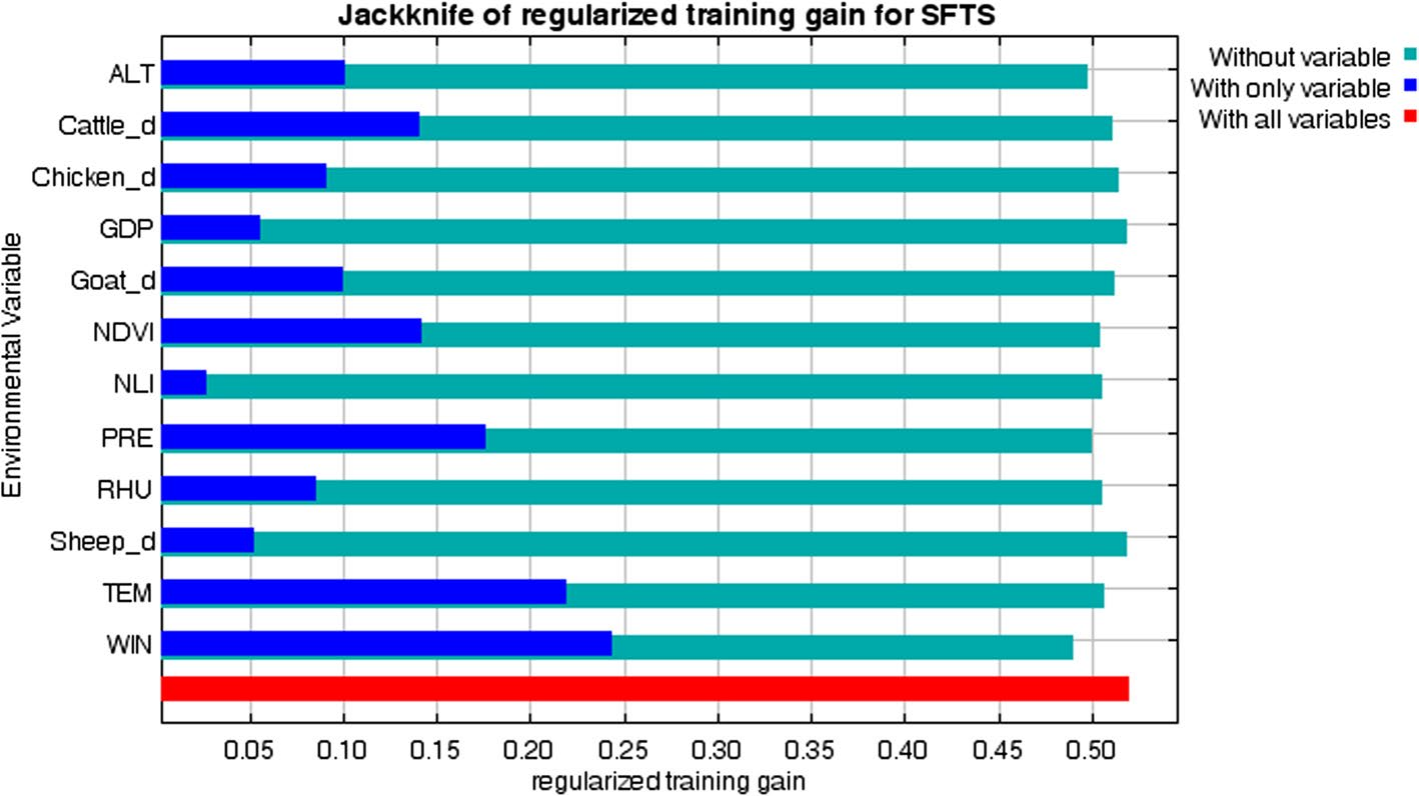
Result of the jackknife test of regularized training gain for SFTS in Shandong Province, China

The AUC value of the model was 0.781, indicating that the model was applicable. The percentage contribution was used to evaluate the relative importance of environmental variables to SFTS occurrence. Among all environmental variables, four variables contributed more than 10% to the model: WIN (mean wind speed, 35.3%), NDVI (normalized difference vegetation index, 14.8%), Cattle_d (cattle density, 12.2%) and PRE (cumulative precipitation, 11.6%), indicating that they were key factors in SFTS occurrence (Table 1). In addition, the jackknife test showed that WIN, PRE, Cattle_d and NDVI all fit the training data well, indicating that they contained the most useful information not contained in the other variables (Fig. 5).

**Fig. 6:**
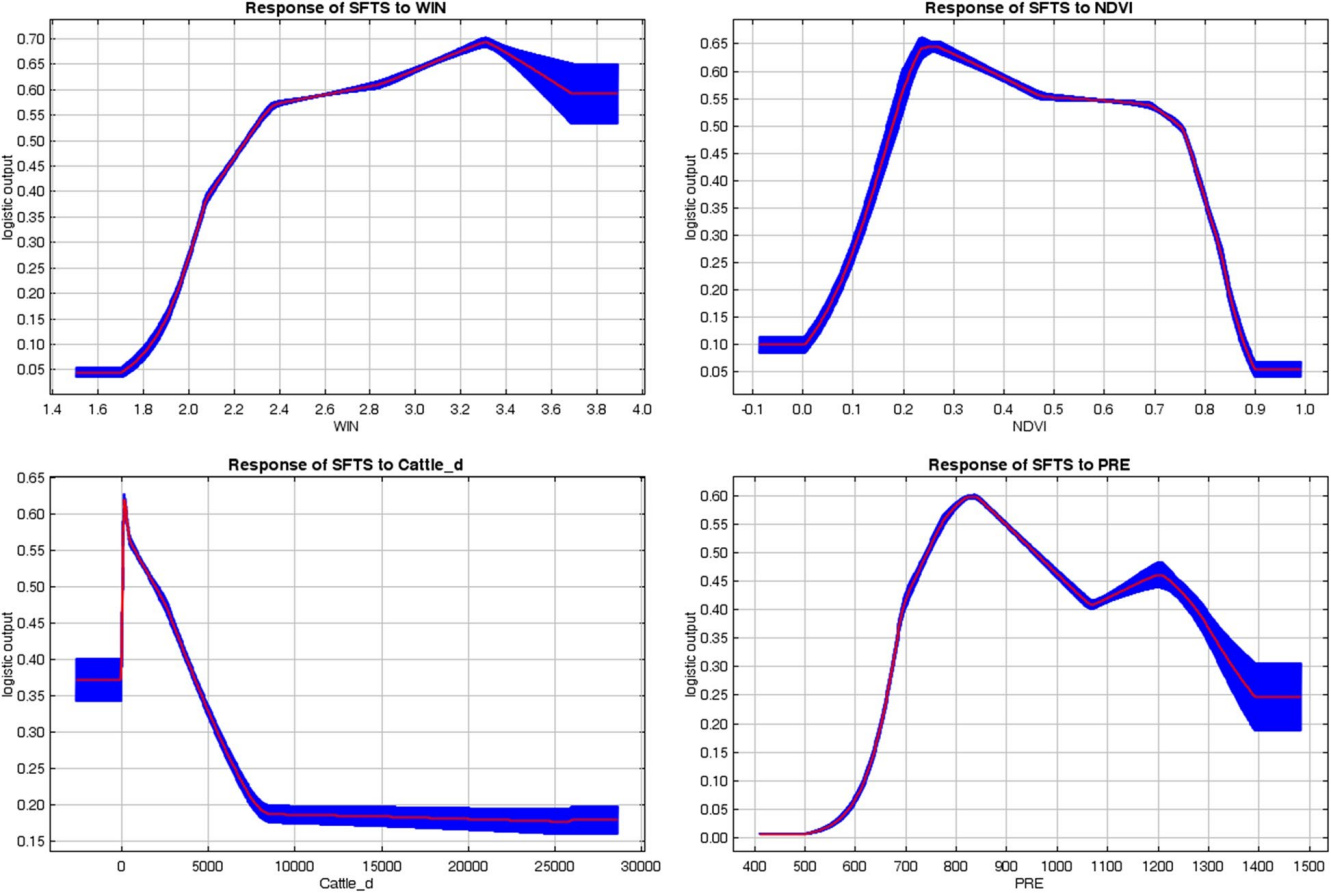
Response curves of SFTS to four significant factors in Shandong Province, China

The response curves for the four significant variables showed that the probability of SFTS occurrence was nonlinearly correlated with the change in each environmental variable. As the wind speed increased, the SFTS occurrence probability started to show an upward trend, peaked at 3.31 m/s, and then decreased. When the wind speed was greater than 2.25 m/s, the probability of SFTS occurrence exceeded 0.50. The response curve of the NDVI showed an inverted U-shape, with an optimal range of 0.18 to 0.75. With the increase in cattle density, the probability of occurrence gradually decreased, and the most suitable cattle density range was 52.06 to 1954.98 cattle/km^2^. The response curve of precipitation showed an inverted V-shape, with an optimal range of 743.88 to 956.05 mm (Fig. 6).

**Fig. 7:**
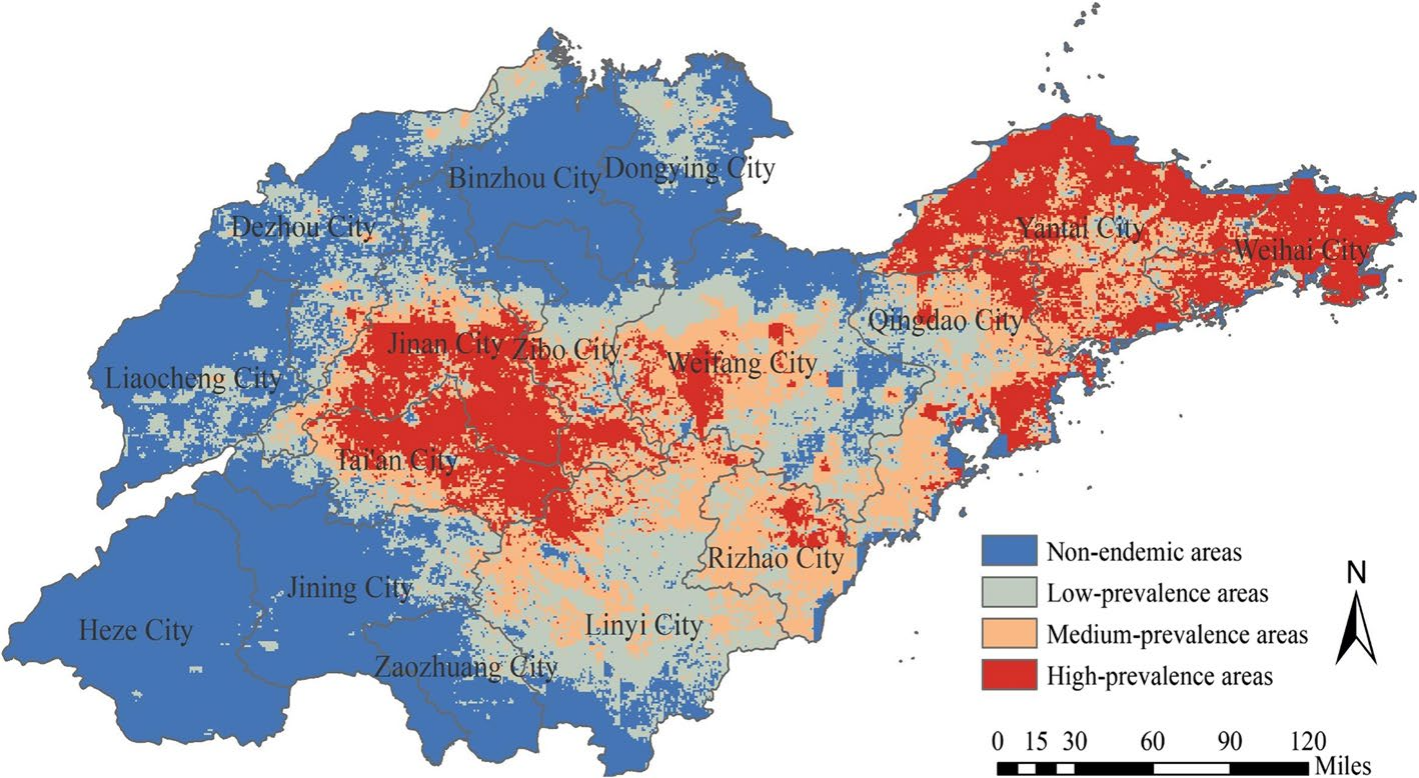
Predicted risk map of SFTS occurrence in Shandong Province, China

The predicted risk map showed that the areas of high-prevalence, medium-prevalence, low-prevalence and nonendemic areas were 28,120 km^2^, 28,900 km^2^, 35,180 km^2^ and 63,600 km^2^, accounting for 18.05, 18.55, 22.58 and 40.82% of the total area of the province, respectively. The high-prevalence areas of SFTS were mainly distributed in the central, eastern and southeastern parts of Shandong Province, while the nonendemic areas were mainly in the western and northern parts. Some cities were obvious high-prevalence areas, mainly including Jinan, Tai’an, Zibo, Weifang, Rizhao, Qingdao, Yantai and Weihai (Fig. 7).

## Discussion

Since Chinese experts first discovered SFTS in 2009, human research on this emerging disease has been carried out for more than 14 years. Based on the early recognition of SFTS, Shandong began to carry out province-wide surveillance, reporting, prevention and control and to report cases according to the national standards for Class B infectious diseases [33]. This study was conducted during the critical period 14 years after the discovery of SFTS in Shandong Province, aiming to comprehensively analyse its spatiotemporal characteristics and environmental influences to provide a precise basis for the development and updating of subsequent prevention and control strategies.

From 2010 to 2021, the number of SFTS cases in Shandong Province showed a continuous upward trend. This upward trend is consistent with those in Zhejiang and Liaoning Provinces in China [34, 35]. The most likely reason for this is that the monitoring, reporting and diagnostic capabilities of SFTS continue to improve as research on SFTS progresses [36, 37]. However, there are also different views; for example, some scholars believe that the migration of ticks and the small animals that carry them may allow SFTSV to expand to more areas and thus lead to more human infections [38, 39]. Similar to the findings of prior studies [13, 16, 40, 41], SFTS cases were mainly recorded from April to October, which may be related to the dynamics of the ticks. *Haemaphysalis longicornis* and *Dermacentor nuttalli* are dominant tick species in Shandong Province, accounting for 75.3 and 24.7% of the collected ticks, respectively [33, 42]. Its adults are active from March to September, peaking in July, which largely corresponds to the time of onset of SFTS cases [43]. Thus, considering the upward trend and seasonality of SFTS, the community should continue to be vigilant about SFTS and tilt prevention and control resources towards the high-incidence period.

The spatiotemporal scan statistic detected three clusters of SFTS located in the central, eastern and southeastern parts of Shandong. Yao Wang et al. performed a similar study on SFTS monitoring data in Shandong prior to 2018 and found that SFTS clustered in the central and eastern parts of Shandong Province [33]. Compared to their study, this study added a new southeastern cluster, suggesting that the SFTS epidemic has expanded into the region. From a spatial perspective, these clusters were all located in the mountainous and hilly areas of Shandong, where tick densities are higher and people are at greater risk of exposure. Kobayashi Y et al. found that SFTS-endemic areas had similar hilly ecological environments, and 82% of infected individuals had visited hills and woodlands before onset [44]. From a temporal perspective, the SFTS clusters occurred in both 2020 and 2021, the last 2 years of our study period, closer to the current period. This indicates that SFTS is becoming more aggregated and that the epidemiological situation is becoming more critical over time.

Various models have been used by previous scholars to study the ecology of SFTS. Zhang et al. used the MaxEnt model to demonstrate that slope and temperature influence the occurrence of SFTS in Jiangsu Province [28]. Sun et al. also used the MaxEnt model and found that the environmental factors affecting the distribution of SFTS in mainland China are altitude, temperature, precipitation and relative humidity [15]. Liu et al. applied Poisson regression analysis and found that the spatial distribution of SFTS in Xinyang city was significantly correlated with the cover of forests, shrubs, and farmland [45]. Based on a generalized linear model, Wang et al. demonstrated that temperature, air pressure and wind speed were associated with the incidence of SFTS in Liaoning Province [35].

In this study, we confirmed that yearly average wind speed, NDVI, cattle density and yearly cumulative precipitation were the most important variables affecting the occurrence of SFTS. Wind speed was positively correlated with the occurrence of SFTS. This may be due to the light weight of the ticks, causing them to be dispersed to various locations by the wind, increasing exposure to humans and other host animals. Areas with a very low NDVI were not conducive to tick survival, and areas with a very high NDVI had almost no footprint of human activity and thus may only have had SFTSV infections when the NDVI was appropriate. The result of Liu et al. was similar to ours, and they found that the area at the junction of cultivated and forested land may be the area with the highest risk of SFTS and that the incidence of SFTS was instead lower in areas with high forest cover [46]. Cattle density was negatively correlated with SFTS cases, which may be due to different feeding practices. In low-density areas, cattle were generally free-range, and in high-density areas, cattle were usually concentrated. A meta-analysis confirmed that SFTSV seropositivity was significantly higher in free-ranging animals than in intensively farmed animals [47]. Precipitation showed a nonlinear relationship with SFTS cases, which one study attributed to the fact that precipitation affects tick populations, with both very high and very low precipitation being detrimental to tick survival [48]. In addition, the predicted risk map showed that the most suitable environment for SFTS infection in Shandong Province was mainly located in the central and eastern regions, so these regions should strengthen prevention and control.

Some limitations of this study should be noted. First, the CISDCP system through which we acquired data was a passive surveillance system, which meant that it was difficult to avoid reporting bias. It is possible that some SFTS patients were not being monitored. Second, it is important to note that the MaxEnt model can only predict conditions in areas with similar conditions using existing factors and thus may deviate from reality. In fact, other factors, such as vector abundance, population density, and soil type may narrow the gap between the predicted and actual SFTS distribution ranges.

## Conclusion

The SFTS epidemic in Shandong Province has a clear upward trend, and its occurrence has obvious seasonality and spatial heterogeneity, with three obvious clusters in the central, eastern and southeastern regions. The occurrence of SFTS is influenced by multidimensional factors, with yearly average wind speed, NDVI, cattle density, and yearly cumulative precipitation as key variables. This contributes to a better understanding of the factors influencing SFTS and provides useful geographic information for public health departments to develop prevention and control strategies. It also suggests that public health departments should incorporate environmental impacts into SFTS prevention and control programs. The modelling approach and results of this study can also be generalized to similar SFTS-endemic areas to implement targeted measures.

### Supplementary Information

**Additional file 1.**

## Data Availability

The datasets used and/or analyzed during the current study are available from the corresponding author on reasonable request.

## Abbreviations

SFTS: Severe fever with thrombocytopenia syndrome
MaxEnt: Maximum entropy
CISDCP: China Information System for Disease Control and Prevention
RR: Relative risk
LLR: Logarithmic likelihood ratio
AUC: Area under the receiver operating characteristic curve
