# Supplementary material for: Spatiotemporal distribution and environmental influences of severe fever with thrombocytopenia syndrome in Shandong Province, China
Source: BMC Infect Dis. 2023 Dec 20;23:891. doi: 10.1186/s12879-023-08899-1 (PMC10731860; doi:10.1186/s12879-023-08899-1)
Supplement: Supplementary file 1 — Additional file 1. [file 12879_2023_8899_MOESM1_ESM.doc]

**Spatiotemporal distribution and environmental influences of severe fever with thrombocytopenia syndrome in Shandong Province, China**

Supplementary Table 1. Spatiotemporal clusters of SFTS cases in Shandong at the county level, 2010–2021.

| Variables | Most likely cluster | 1st Secondary clusters | 2nd Secondary clusters |
| --- | --- | --- | --- |
| Longitude (E) | 121.36 | 117.64 | 119.41 |
| Latitude (N) | 37.52 | 36.30 | 36.01 |
| Radius (km) | 125.81 | 76.44 | 85.44 |
| Time frame | 2021/5 to 2021/10 | 2021/5 to 2021/10 | 2020/5 to 2020/9 |
| Population | 10578064 | 13356130 | 12042590 |
| Number of counties | 18 | 19 | 15 |
| Cluster counties | Laixi, Zhifu, Fushan, Muping, Laishan, Changdao, Longkou, Laiyang, Laizhou, Penglai, Zhaoyuan, Qixia, Haiyang, Kaifaqu, Huancui, Wendeng, Rongcheng, Rushan | Lixia, Shizhong, Licheng, Changqing, Zhangqiu, Laiwu, Gangcheng, Gaoxin, Zichuan, Zhangdian, Boshan, Zhoucun, Yiyuan, Gaoxin, Taishan, Daiyue, Xintai, Mengyin, Zouping | Huangdao, Jiaozhou, Weicheng, Fangzi, Kuiwen, Linqu, Changle, Zhucheng, Anqiu, Gaomi, Donggang, Lanshan, Wulian, Ju, Yishui |
| Annual cases/100,000 | 8.40 | 4.40 | 1.50 |
| Observed/expected | 17.33 | 9.02 | 3.17 |
| Relative risk | 18.71 | 9.47 | 3.20 |
| Loglikelihood ratio | 865.80 | 400.89 | 38.28 |
| *p* value | ＜0.01 | ＜0.01 | ＜0.01 |
